# Field Epidemiology in Africa

**Published:** 2011-12-14

**Authors:** Sheba Nakacubo Gitta, Fred Wabwire-Mangen, Mufuta Tshimanga

**Affiliations:** 1 African Field Epidemiology Network (AFENET); 2 Makerere University School of Public Health, School of Public Health, Makerere University, Kampala, Uganda; 3 Zimbabwe Field Epidemiology Training Program, Department of Community Medicine, Harare, Zimbabwe

**Keywords:** Africa, field epidemiology, capacity building, diseases surveillance, networks, zoonoses

## Editorial

Field epidemiology involves the application of basic epidemiology principles in real time, at a specific place, and on specific people to solve public health problems especially emergencies. Africa has several opportunities for public health officials to practice field epidemiology as there are frequent disease outbreaks and other public health emergencies. However public health systems in Africa are implemented jointly with health care delivery; there are very few public health professionals (especially physicians) who do not play dual roles of health care delivery and public health practice.

Public health surveillance has been defined as the ongoing systematic collection, analysis, interpretation, and timely dissemination of health-related data to those who are able to take public health action. This definition implies a separation between the public health surveillance workforce and the public health response workforce. However, following the adoption and widespread use of the World Health Organization Regional Office for Africa's Integrated Disease Surveillance and Response (IDSR) strategy, public health surveillance and public health response in Africa are often conducted by personnel, who also perform routine health care, and are therefore overworked. A further complexity to the practice of field epidemiology in Africa is the weakness of the public health (and clinical) laboratory networks which are critical for effective public health surveillance especially for communicable diseases. Additionally, diseases that cross the animal human interface are common and devastating viral hemorrhagic fevers and other zoonoses are frequent in African countries. Clearly a strategy to develop a public health workforce in Africa which can practice field epidemiology in Africa should include joint postgraduate training of physicians and other health scientists, with public health laboratory scientists and veterinarians. This is likely to strengthen public health surveillance and response in Africa efficiently. It will also augment the current paradigm shift towards ‘One health’ practice which calls for building inter-disciplinary bridges across the human, animal and environmental health sectors to attain optimal health in a globalised world [1].

Several authors have previously described the lack of health care workers in Africa, particularly the lack of clinical care workers. A few have described the lack of public health workers and pointed out that the unmet need for public health workers is likely smaller than the need for health care workers. In 2008 Scheffler and others estimated that the need for physicians was about 0.55 per 1000 population which translates into 550 physicians per million-population [2]. Other authors have posited the need for field epidemiologists to range from 3 to 5 per million-population for a country in sub-Saharan Africa [3]. This is a 2 log order of magnitude less than the physician need and may signify an important opportunity for quick progress. Some authors have shown that graduates of field epidemiology training programs in Africa have a 5-year retention of up to 80% [4]. Many graduates of field epidemiology training programs in Africa have gone on to run important Ministry of Health programs because of the nature of their “in-country in-context” training and the fact that all these programs award a postgraduate degree to successful graduates which is necessary for an advancing career path. Putting all these issues together it is possible to suggest that: a) training field epidemiologists in Africa is a smaller task than developing a health-care workforce and b) this training may lead to sustained results for a workforce that is able to implement multi-disease public health surveillance and response holistically. This does not mean that it is easy, or cheap, or that it is more important than developing the health-care workforce but that it may be more manageable.

The authors of the papers in this special PAMJ-African Field Epidemiology Network (AFENET) 5^th^ year anniversary supplement have described how different field epidemiology and laboratory training programs have been developed in several countries with unique country-led and country-owned partnerships. They have also described how these programs have trained physicians and other health scientists, with public health laboratory scientists, and in some cases with veterinarians, in field epidemiology with the intent of using the trainees and graduates to implement and lead multi-disease surveillance and response systems. Other authors have described the development and implementation of AFENET which is a regional service and networking alliance of these programs, based on a novel partnership between training institutions and ministries of health. There is a paper on the early results of the public health laboratory graduates from the programs and there are papers on the “One Health” training which includes physicians, health scientists, public health laboratory scientists, and veterinarians. Some of the papers in this supplement have been written by trainees and graduates from field epidemiology and laboratory training programs in Africa. They showcase examples of trainee outputs and give credence to the successes described in the different program papers. The authors have been candid in the challenges they face as they implement field epidemiology and laboratory training programs and the network to link them. It is our hope that this supplement enables the reader to learn about the unique nature of the training and practice of field epidemiology in the African context, knowing that these programs will underwrite a significant portion of global health security as the graduates of these programs will be the ones to implement public health surveillance and response in the context of the revised International Health Regulations and IDSR.
